# A Prospective Observational Study to Evaluate Cardiovascular Changes in Patients of Hypothyroidism

**DOI:** 10.7759/cureus.40201

**Published:** 2023-06-09

**Authors:** Priyanka Sawarthia, Deepak Bhosle, Ravi Kalra

**Affiliations:** 1 Internal Medicine, Bharati Vidyapeeth Deemed to be University, Pune, IND; 2 Internal Medicine, Bharati Vidyapeeth (Deemed to be University) Medical College, Pune, IND; 3 Cardiology, Bharati Vidyapeeth (Deemed to be University) Medical College, Pune, IND

**Keywords:** hypothyroidism, cardiovascular disease, 2d echo, 12-lead ecg, ecg, functional hypothyroidism

## Abstract

Introduction

Hypothyroidism is a common endocrine disorder in India and is easy to diagnose based on clinical manifestations and signs. Thyroid hormone affects the cardiovascular system. Fatiguability, dyspnea, weight gain, lower limb swelling, and bradycardia are some clinical manifestations. ECG changes in hypothyroidism include sinus bradycardia, prolonged QTc interval, changes in the morphology of the T-wave, QRS duration, and low voltage. Echocardiography changes include diastolic dysfunction, asymmetrical septal hypertrophy, and pericardial effusion. This study aimed to examine the cardiovascular changes in patients with hypothyroidism.

Methodology

Patients with hypothyroidism and cardiovascular changes were assessed using an electrocardiogram and echocardiography.

Results

A total of 68 hypothyroid patients were enrolled in the study. The mean age of patients was 41.93 ± 15.36 years, and the mean BMI was 24.64 ± 4.30 kg/m^2^. Of 68 hypothyroid patients, 57 (83.8%) were females, and 11 (16.2%) were males. The mean thyroid-stimulating hormone (TSH) level in the study population was 11.48 ± 22.02 (mIU/mL). The most common symptoms reported among the study participants were tiredness or weakness (67.6%), followed by dyspnea (42.6%). The mean pulse rate, systolic blood pressure, and diastolic blood pressure were 81.50 ± 16.16, 112.76 ± 7.05, and 70.68 ± 7.46, respectively. Pallor was the most common sign (22.1%) among all the people who participated in the study. The most common findings on the ECG were low voltage complexes (25%) followed by inversion of the T wave (23.5%). Other ECG findings were bradycardia (10.3%), right bundle branch block (7.4%), and QRS prolongation (2.9%). Echocardiography revealed 21 (30.8%) patients with grade 1 left ventricular diastolic dysfunction and pericardial effusion in two patients (2.94%). There was a significantly greater increase in the level of TSH in study participants.

Conclusion

Patients with abnormal ECG and echocardiography without other cardiovascular changes should be evaluated for hypothyroidism to improve the quality of care.

## Introduction

Thyroid disorders are among the most common endocrine disorders in the world. India is not an exception. According to estimates based on various studies, thyroid disease affects approximately 42 million people in India. In the adult Indian population, it is a very common disorder. It affects one out of every 10 patients [1]. It is defined as low levels of serum thyroid hormone caused by the destruction of the thyroid gland. Autoimmunity or interventions such as surgery, drugs, iodine deficiency, or radiation are the most common causes of destruction [2]. Fatiguability, muscle weakness, cold intolerance, hair loss, difficulty concentrating and memory, dyspnea, hoarse voice, menorrhagia, constipation, weight gain, paresthesia, and impaired hearing are some of the clinical manifestations. Dry and coarse skin, cool peripheral extremities, puffy face, hands, and feet, diffuse alopecia, bradycardia, peripheral edema, delayed tendon reflexes, carpal tunnel syndrome, and serous cavity effusions are just a few of the clinical signs [3]. Overt hypothyroidism is defined as a condition in which the levels of thyrotropin (thyroid-stimulating hormone) are high and serum T4 (free thyroxine) is low [4]. Thyroid hormones profoundly affect several metabolic processes in virtually all tissues. Hence, thyroid hormone deficiency affects every tissue to some degree, with the heart being particularly sensitive to its effects. Hypothyroid patients have a higher risk of functional cardiovascular abnormalities, which increases their cardiovascular risk [5]. Hypothyroidism causes a rise in diastolic blood pressure due to increased systemic vascular resistance. The heart's contractility and output are reduced, resulting in a narrowed pulse pressure. In overt hypothyroidism, left ventricular dysfunction is a common finding. It is defined as abnormal diastolic relaxation or distensibility of the left ventricle, regardless of whether the ejection fraction of the left ventricle is normal or abnormal and whether or not the patient is symptomatic [6]. One of the most important factors is altered intracellular calcium handling. Hypothyroidism causes left ventricular hypertrophy and diastolic dysfunction due to increased body weight and blood pressure [7]. Furthermore, increased intramyocardial fibrosis may play a role in overt hypothyroidism. After treatment, diastolic dysfunction can be reversed [8]. Other cardiovascular risks linked to hypothyroidism include increased serum cholesterol, C-reactive protein, and homocysteine, which can lead to accelerated atherosclerosis, stroke, and coronary artery disease. Bradycardia, low voltage complex, right bundle branch block (RBBB), QT prolongation, and T wave inversion are all ECG changes seen in hypothyroidism [9]. There is also QRS prolongation, RBBB, and Torsades de Pointes on rare occasions [10]. Diastolic dysfunction, pericardial effusion, and asymmetrical septal hypertrophy are the most common echocardiographic changes [9].

The current study was designed to evaluate various cardiovascular changes in hypothyroidism patients using clinical findings, electrocardiography, and echocardiography.

## Materials and methods

This prospective observational study was conducted at a tertiary health care center (Bharati Vidyapeeth Deemed to be University Medical College Hospital) for 18 months from August 2021 to January 2023. The institutional ethical committee's permission was obtained (Bharati Vidyapeeth Deemed to be University Medical College, Pune; reference number: BVDUMC/IEC/02), and patient consent was taken before sample collection.

A total of 68 adult patients with hypothyroidism were included in this study. All patients aged > 18 years presenting to the medicine/endocrine OPD and inpatient department of Bharati Hospital who were newly diagnosed or known cases of hypothyroidism according to thyroid function test were included in the study after taking written informed consent. Patients aged less than 18 years, with a history of cardiac disease, diabetes mellitus, and hypertension, or who refused to give consent were excluded from this study.

A pre-structured proforma was used to collect the patient's baseline characteristics and detailed history. A detailed general physical examination and cardiovascular examination were carried out. Complete blood count, blood sugar profile, renal function test, and urine routine were also carried out. Electrocardiography was done in all patients. ECG findings such as low voltage complex, inversion of the T wave, bradycardia, RBBB, and QRS prolongation were assessed by two independent investigators. Patients were assessed echocardiographically by standard M mode on a GE Vivid E95 machine. Two-dimensional (2D) echo findings such as ejection fraction (EF) %, E wave, A wave, E’, E/A ratio, E/E’ ratio by tissue Doppler imaging, left-ventricular diastolic dysfunction, S/D ratio, deceleration time, and left atrial (LA) volume were measured. All parameters were assessed by the principal investigator and expert cardiologist.

The collected data were coded and entered into the Microsoft Excel sheet (Microsoft Corporation, Redmond, WA). The data were analyzed using STATA version 15 software (StataCorp LLC, College Station, TX) [10]. For quantitative data, various rates, ratios, and percentages were calculated. For quantitative data, mean/median and SD were calculated. A p-value of less than 0.5 was considered statistically significant.

## Results

The present study was carried out to assess the cardiovascular changes concerning ECG and echocardiographic findings in patients of hypothyroidism, along with the cardiovascular clinical profile. A total of 68 hypothyroid patients were enrolled in the study. The mean age of the patients was 41.93 ± 15.36 years, between 18 and 94 years, and the mean BMI was 24.64 ± 4.30 kg/m^2^, between 16.5 to 40.5 kg/m^2^. The majority of patients were below the age of 40 years, 19 (27.9%) were below the age of 30 years, 20 (29.4%) were between the age group of 31 and 40 years, followed by 11 (16.2%) between the ages of 41 and 50 years and 18 (26.5%) above the age of 50 years. Of 68 hypothyroid patients, 57 (83.8%) were females, and 11 (16.2%) were males.

The most common symptom reported among the study participants was tiredness/weakness (67.6%). Other symptoms were dyspnea (42.6%), hair loss (41.2%), cold intolerance (35.3%), weight gain with poor appetite (33.8%), dry skin (23.5%), and paresthesia (20.6%). Other uncommon symptoms were constipation (11.8%), difficulty concentrating and poor memory (10.3%), menorrhagia (10.3%), and hoarseness of voice (4.4%). The pallor was the most common (22.1%) sign reported among all the study participants, followed by edema seen in two (2.9%) and lymphadenopathy in only one (1.5%) patient. The mean pulse rate was 81.50 ± 16.16, systolic blood pressure (SBP) was 112.76 ± 7.05, and diastolic blood pressure (DBP) was 70.68 ± 7.46.

Table 1 shows the biochemical parameters, including thyroid profile and hematological parameters, estimated in these populations. The mean thyroid-stimulating hormone (TSH) level in the study population was 11.48 ± 22.02 (mIU/ml).

**Table 1:** Biochemical and hematological laboratory findings

Parameter	Mean ± SD
Hemoglobin	11.10 ± 2.03
Total leukocyte count	8473.24 ± 3385.04
Platelet count	3296.50 ± 15823.21
Blood sugar level - random	95.88 ± 15.71
Serum T3 (ng/dL)	3.96 ± 17.04
Serum T4 (mcg/dL)	6.06 ± 3.14
Serum thyroid-stimulating hormone (mIU/ml)	11.48 ± 22.02

The most common findings on ECG were low voltage complex (n = 21, 30.9%), followed by inversion of the T wave (n = 16, 23.5%). Other ECG findings were bradycardia (n = 7, 10.3%), RBBB (n = 5, 7.4%), and QRS prolongation (n = 2, 2.9%). Normal ECG was seen in 25% of participants.

Echocardiography findings suggest grade I diastolic dysfunction was found in 30.8% of study participants, and pericardial effusion was reported in 2.94%. The levels of TSH, according to the ECG findings, were compared between the groups. There was a significant increase in the level of TSH in patients with low voltage complex than those without. While for other parameters evaluated, no significant difference was found between the groups. Table 2 shows the median (interquartile ranges) of TSH values per the ECG findings.

**Table 2:** Association of serum TSH as per ECG findings * Statistically significant. TSH: thyroid-stimulating hormone; RBBB: right bundle branch block.

Parameter	Yes/No	N	Median (IQR)	p-value
Bradycardia	Yes	7	2.86 (2.38-5.95)	0.62
	No	61	4.63 (2.22-8.15)	
Low voltage complex	Yes	21	5.46 (3.58-43.91)	0.021*
	No	47	4 (1.61-5.99)	
RBBB	Yes	5	5.95 (1.66-8.12)	0.87
	No	63	4.36 (2.32-8.12)	
Inversion of the T wave	Yes	16	3.42 (0.78-5.93)	0.146
	No	52	4.71 (2.38-8.17)	
QRS prolongation	Yes	2	4.02 (4-4.02)	0.76

## Discussion

Cardiovascular disease is the leading cause of death worldwide. Traditional risk factors like hypertension, dyslipidemia, and diabetes significantly contribute to the emergence of cardiovascular disease. An important reduction in the risk of cardiovascular events is linked to the intensive treatment of these risk factors. The fact that many patients still have a sizable residual risk even after addressing these risk factors emphasizes the significance of comprehending additional mechanisms underlying cardiovascular dysfunction [11]. One of the most profound and clinically significant findings that accompany both hyperthyroidism and hypothyroidism are the cardiovascular signs and symptoms of thyroid disease [9,12,13].

Thyroid hormones significantly influence the development and homeostasis of the cardiovascular system [14-16]. Hypothyroidism is a common endocrine condition caused by a lack of thyroid hormone [9]. Both overt and subclinical thyroid dysfunction are frequently seen in the general population. They are closely linked to the cardiovascular system and impact the hemodynamics, cardiac mass, cardiac contractility, and autonomic control of the cardiovascular system [17]. They are also linked to higher morbidity and mortality rates from all causes and higher rates of cardiovascular mortality [17-19]. Thyroid hormones may modulate the risk of atherosclerosis development and have electrophysiologic and inotropic effects on the heart [20]. Previous studies demonstrated that patients with diagnosed hypothyroidism, even those with subclinical forms of the condition, had both diastolic and systolic dysfunction [21-24].

Of 68 hypothyroid patients, 57 (83.8%) were females, and 11 (16.2%) were males, enrolled in the study between 18 and 94 years. The most common age group was between 31 and 40 years. The mean BMI ranged from 16.50 to 40.50 kg/m^2^. The means of T3 (0.58-1.59 ng/dL), T4 (4.87-11.72 mcg/dL), and TSH (0.40-4 mIU/ml) levels in the study population were 3.96 ± 17.04, 6.06 ± 3.14, and 11.48 ± 22.02, respectively. According to a study by Ramesh et al. [24], most cases affected people between 31 and 40 years. The average age was 35 years. In their study, approximately 75% of the total population were women. In a study by Shrivastava et al. [9], 90 patients were included. The results were similar to those of the current study in that 90% of the participants were female patients, compared to 83.8% in the current study. The average age of the 72 patients with thyroid dysfunction evaluated by Satpathy et al. [25] ranged from 15 to 65 years. The third and fourth decades saw the highest incidence of thyroid dysfunction. The most prevalent symptoms, according to Ramesh et al. [24], were weight gain (72.5%), lethargy (65%), and dry skin (62.5%). The present study reported that the most common symptoms were tiredness or weakness (67.6%). Other symptoms were dyspnea (42.6%), hair loss (28%), cold intolerance (24%), weight gain with poor appetite (23%), dry skin (16%), paraesthesia (16%), menorrhagia (10.3%), and constipation (8%). Amongst the sign, pallor was the commonest sign (22.1%).

Different ECG changes are linked to the range of thyroid dysfunction, and they may be crucial because even minor ECG changes have prognostic significance. Ramesh et al. [24] reported that in their study, 30% of the patients had normal ECGs, with bradycardia present in 40% of cases and low voltage complexes in 30%. A study by Shende et al. [26] reported that the low-voltage complex (48.9%) was the most typical ECG finding, followed by RBBB. ECG changes reported by Shrivastava et al. [9] were sinus bradycardia (35.5%), low voltage complexes (16.6%), T-wave inversion (7.8%), RBBB (4.4%), and QTc prolongation (2.2%), which were most similar to the findings of the present study. According to Saxena et al. [27], 57% of hypothyroid cases had abnormal ECG results. The most frequent ECG abnormality was bradycardia, affecting 27% of patients. Of the patients, 25% exhibited low voltage complexes. Patients with bradycardia and a low-voltage complex showed up in 5% of cases. In a study by Satpathy et al. [25], the most frequent ECG finding in 34% of hypothyroid patients was a horizontal ST segment depression with a T wave inversion. All patients with ST-T changes had stable angina, while the second most common finding was sinus bradycardia, seen in 23% of patients.

In the present study, 75% of the hypothyroid patients were found to have changes in their ECG. However, 25% of study subjects had normal sinus rhythm. The most common ECG finding was low voltage complex (30.9%). Others were the inversion of the T wave (23.5%), bradycardia (10.3%), RBBB (7.4%), and QRS prolongation (2.9%). No regional wall motion abnormality was seen in the 2D echo of patients who had T wave inversions in the ECG. The levels of TSH, according to the ECG findings, were compared between the groups. There was a significantly greater increase in the level of TSH in patients with low-voltage complexes than in those without.

Pericardial effusion was observed in 11 cases (27.5%), and according to Ramesh et al. [24], 27.5% of patients had diastolic dysfunction, the majority of which was mild. There were no cases of severe diastolic dysfunction. Saxena et al. [27] reported abnormal findings in 38% of hypothyroid patients, the most frequent of which was mild pericardial effusion (18%). Satpathy et al. [26] reported significantly lower ejection fraction (EF) values in hypothyroid patients compared with hyperthyroid subjects. Even though the patients had subclinical hypothyroidism and autoimmune thyroiditis, Zoncu et al. [28] found problems with the heart's systolic and diastolic functions. According to Shrivastava et al.'s [9] findings from an echocardiographic examination, changes were present in about 64% of cases. Diastolic dysfunction was the most frequent finding, occurring in 20% of cases, followed by pericardial effusion (16.6% of cases), increased interventricular septal thickness (IVSD) (16.6%), and increased left ventricular posterior wall (LVPW) (11.1%). According to Hadzović-Dzuvo et al. [29], the average E/A value, a measure of diastolic function, was significantly lower in the hypothyroid group compared to the hyperthyroid and euthyroid groups. However, after treatment, the E/A values increased in patients with hypothyroidism. In the present study, according to 2D echocardiography findings, grade I diastolic dysfunction was found in 30.8% of study participants, and pericardial effusion was reported in 2.94%.

Limitations

The limitations of this study are the small sample size, patients coming into the hospital cannot be generalized to the general population, as patients with subclinical hypothyroidism or with symptoms such as fatigue can be missed, and selection bias.

## Conclusions

Hypothyroidism presents in many forms. Patients with abnormal ECG and echocardiography in the absence of other cardiovascular changes should be evaluated for hypothyroidism to improve the quality of care.
